# NBPF7 promotes the proliferation of α-catenin-knockdown HaCaT cells via functional interaction with the NF-κB pathway

**DOI:** 10.18632/oncotarget.19480

**Published:** 2017-07-22

**Authors:** Hua-Yu Zhu, Wen-Dong Bai, Chao Li, Jun Li, Da-Hai Hu

**Affiliations:** ^1^ Department of Burns and Cutaneous Surgery, Xijing Hospital, Fourth Military Medical University, Xi’an, Shaanxi, P.R. China; ^2^ Clinical Laboratory Center, Xinjiang Command General Hospital of Chinese People’s Liberation Army, Urumqi, Xinjiang, P.R. China; ^3^ Center of Military Burns and Plastic Surgery, Lanzhou General Hospital of Lanzhou Military Command of Chinese People’s Liberation Army, Lanzhou, Gansu, P.R. China

**Keywords:** α-catenin, NBPF7, NF-κB, HaCaT, proliferation

## Abstract

Loss of key components that form cell-cell adherens junctions, such as α-catenin, triggers severe epidermal hyperproliferation. However, the underlying molecular mechanisms remain largely unknown. We report here that neuroblastoma breakpoint family (NBPF) genes are upregulated and that NBPF7 specifically promotes cellular proliferation of α-catenin-silenced HaCaT cells through functional linkage with the NF-κB pathway. Genome-wide profiling of HaCaT cells shows that NBPF genes are upregulated following α-catenin knockdown. Data from western blot analyses are consistent with the activation of the NF-κB pathway as well as increased expression of NBPF7 by α-catenin knockdown. Co-immunoprecipitation assays indicate that NBPF7 could be detected in endogenous activated NF-κB immunoprecipitates. Immunoflurence analyses demonstrate that NBPF7 co-localizes with activated NF-κB in the nucleus after α-catenin silencing. Moreover, inhibition of NBPF7 decreases the proliferation of HaCaT cells and abolishes the enhanced proliferation associated with α-catenin knockdown in HaCaT cells. These results indicate that NBPF7 plays a key role in the α-catenin signaling pathway that regulates cell proliferation of keratinocytes. Our findings suggest that the classical NF-κB pathway plays a critical role in cellular proliferation and that NBPF7 is a functional mediator for α-catenin in the regulation of keratinocyte growth.

## INTRODUCTION

The actin-binding protein α-catenin (α-cat, CTNNA) plays a crucial role in establishing intercellular adhesion, regulating cortical tension, and maintaining mechanical coupling between cells [1, 2]. During classical cadherin binding, cadherin adhesion receptors are recruited to α-catenin and function to anchor the cadherin-catenin complex to the actin cytoskeleton at adherens junctions [3-5]. A recent study demonstrated that α-catenin is one of the growing list of actin-binding proteins that can also modulate gene transcription, possibly by controlling the dynamics of actin in the nucleus [6]. An increasing body of evidence implicates the downregulation of α-catenin in the activation of different signaling pathways that promote nuclear localization of nuclear factors; thereby, underscoring the importance of unraveling the underlying cellular and molecular mechanisms [7-9]. Similar to the loss of tumor suppressor genes such as TP53, loss of-α-catenin function results in increased cell proliferation both *in vivo* and *in vitro* [10, 11]. A previous study showed that knockdown of α-catenin in a HaCaT cell line resulted in a hyperproliferative phenotype in keratinocytes [7]. The underlying molecular mechanism, however, remains largely unknown.

The nuclear factor κB (NF-κB) is a nuclear transcription factor that regulates the expression of a large number of genes critical for cell proliferation, apoptosis, survival, viral replication and inflammation. Indeed, dysregulation of NF-κB has been found in several types of cancers and autoimmune diseases [12, 13]. Moreover, members of the neuroblastoma breakpoint family (NBPF) of genes are highly expressed in a variety of tissues and cell types, including embryonic stem cells, fetal and adult tissues, and normal and cancerous tissues [14-16]. Indeed, the NBPF family of genes is densely covered by many high-confidence ChIP-Seq peaks of NF-κB [17]. However, the identity of the exact genes of interest and their putative biological functions in α-catenin-knockdown keratinocytes cells remain unknown.

In the present study, we genetically silenced α-catenin in a HaCaT cell model and performed genome-wide analysis of the gene expression profile of these cells. We found that NBPF genes were indeed upregulated. We further investigated the functional linkage between NBPF7 and the NF-κB pathway in the proliferation of α-catenin-knockdown HaCaT cells.

## RESULTS

### NBPF genes are upregulated in α-catenin-knockdown HaCaT cells

**Figure 1:**
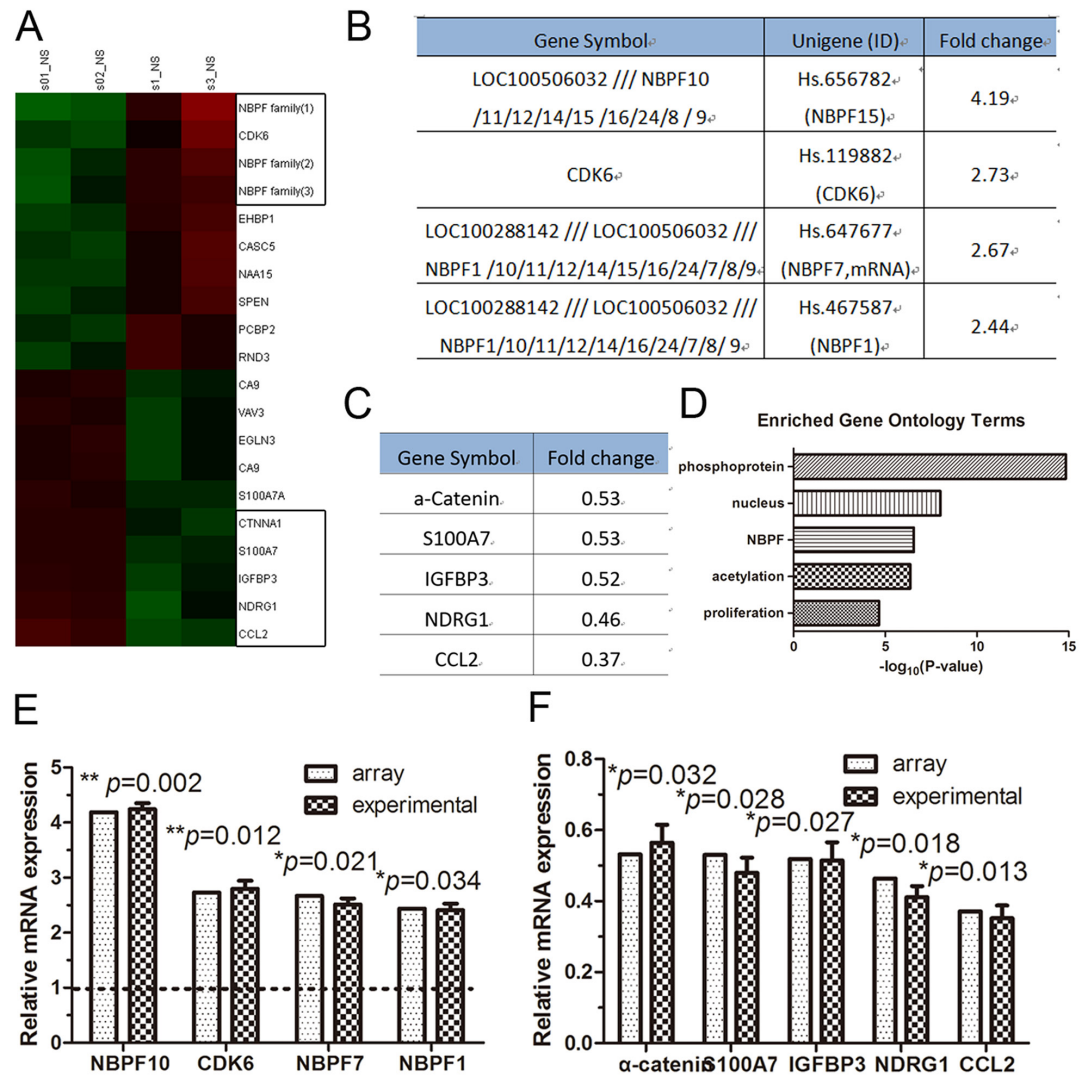
The expression of NBPF genes was increased in α-catenin-knockdown HaCaT cells **(A)** Heat map of the genes that are significantly altered after transient transfection with shRNA (α-catenin) oligonucleotides. **(B)** The RNA level of increased genes. **(C)** The RNA level of decreased genes. **(D)** Gene ontology classification of the up-regulated gene in α-catenin-knockdown HaCaT cells using the DAVID website. **(E)** and **(F)** qRT-PCR analysis of the gene expression levels in α-catenin-knockdown HaCaT cells. Data are presented as means ± SEM (*p*< 0.05, *p*< 0.01, independent t test).

A recent study demonstrated that α-catenin modulates gene transcription by controlling actin dynamics in the nucleus [6]. To identify the genes that may be differentially expressed following silencing of α-catenin with short hairpin RNA (shRNA), we first tested the efficacy of α-catenin knockdown by RNA oligonucleotides (RNA-oligo) in cultured human HaCaT keratinocytes. We found that the mRNA levels of α-catenin were significantly downregulated by α-catenin-1254, which was selected for further experiments (Supplementary Figure 1). HaCaT cells were transfected with α-catenin-1254 or a negative control for 72 hr and subjected to mRNA microarray analysis (Figure 1A). α-catenin-knockdown HaCaT cells displayed significantly higher levels of several members of the NBFP family of genes (Figure 1B). Meanwhile, of the genes that exhibited reduced expression, α-catenin was the 5th most downregulated gene (Figure 1C). It was reported that transient transfection can exhibit transient inter-assay variation and protein-protein interactions due to α-catenin loss and avoid a long-term effect caused by disordered signal transduction pathways [18], thus we used transient transfection samples for genome-wide gene expression analysis for this study. Further gene ontology analysis using DAVID bioinformatics resources revealed that the candidates were functionally enriched in several biological processes, including NBPF family, proliferation, and nucleus processes (Figure 1D). We found several up- and down-regulated gene in α-catenin-knockdown HaCaT cells vs. control cells and we further confirmed their expression using qRT-PCR (Figure 1E and 1F).

### NBPF7 interacts with NF-κB in α-catenin-knockdown HaCaT cells

**Figure 2:**
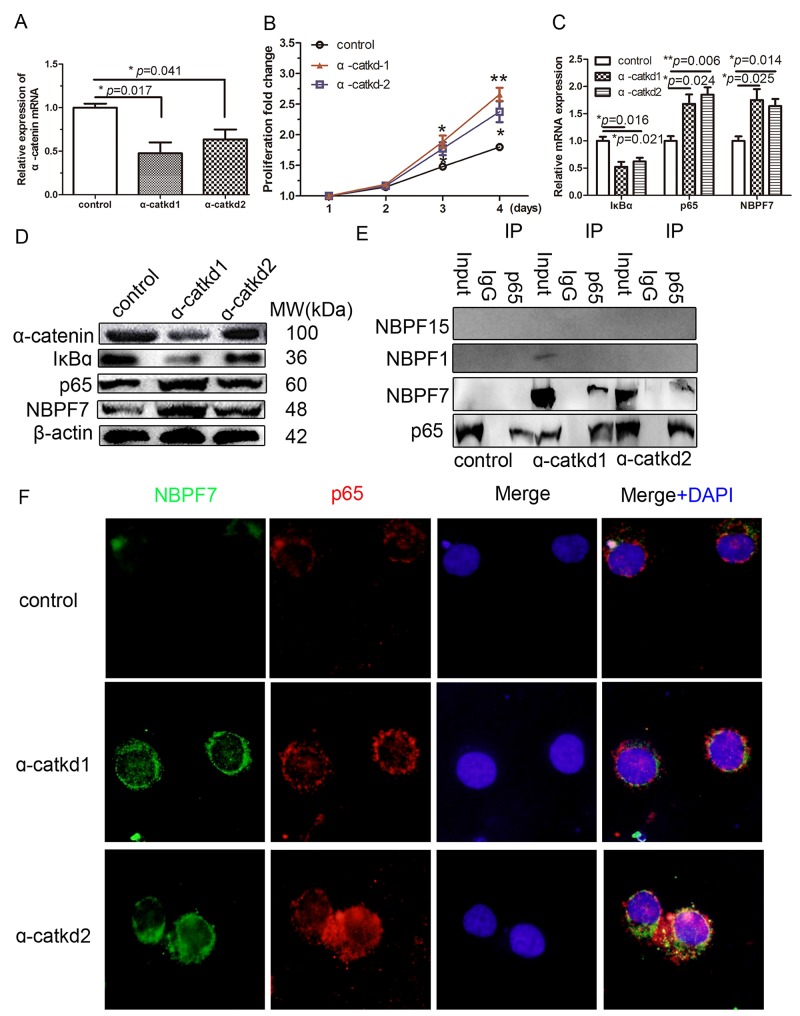
Activated NF-κB pathway interacted with NBPF7 **(A)** α-catenin mRNA in α-catenin-stable knockdown HaCaT cells. **(B)** Stable knockdown of α-catenin resulted in hyperproliferation of HaCaT cells. The mRNA **(C)** and protein **(D)** level of the indicated gene in α-catenin-stable knockdown HaCaT cells. **(E)** Confluent α-catenin-knockdown HaCaT cell lysates (input) were used for an IP with an antibody against p65. The immunoprecipates were blotted with an antibody against NBPF7. NBPF7 associated with p65 in α-catenin-knockdown HaCaT cells. The association of NBPF7 and p65 was diminished in the absence of α-catenin-knockdown, not NBPF1 and NBPF15. **(F)** Immunofluorescence of NBPF7 and p65 in α-catenin-knockdown in HaCaT cells. Data are presented as means ± SEM (*p*< 0.05, *p*< 0.01, independent t test).

To validate the functions of α-catenin, we used 2 lentivirus-mediated shRNAs to decrease endogenous α-catenin levels in HaCaT cells. Quantitative RT-PCR measurements (Figure 2A) revealed that the lentivirus-delivery system (α-catkd1 and α-catkd2) significantly decreased α-catenin mRNA expression as compared to control cells. Consistent with the decrease in mRNA levels, western blot analyses also showed reduced protein levels by α-catkd1 and α-catkd2 (Figure 2D). These results indicate that two selected α-catenin shRNAs, especially α-catkd1, could effectively decrease the mRNA and protein levels. We next assessed potential changes in cell proliferation following α-catenin knockdown in HaCaT cells *in vitro* and found that stable knockdown of α-catenin resulted in enhanced cell growth compared with control cells (Figure 2B). It demonstrates that α-catkd1 enhanced the level of proliferation (Figure 2B). Previously, it was shown that α-catenin regulates the NF-κB response genes in a breast cancer cell line [11]. Our current results suggested that upregulation of the NF-κB pathway might contribute to the enhanced cell proliferation of α-catenin-knockdown HaCaT cells. Indeed, downregulation of α-catenin markedly suppressed the protein level of IκBα and increased the p65 protein level, indicating that one of the mechanisms by which α-catenin promotes cell growth is to increase the activity of the NF-κB pathway.

We next asked whether α-catenin-mediated upregulation of p65 involves a physical interaction with NBPF. To address this, we performed co-immunoprecipitation (Co-IP) assays with a NBPF antibody that can recognize most NBPF members (see Materials and Methods). We found that NBPF7 could be detected by the NBPF antibody in endogenous p65 immunoprecipitates from the α-catenin-knockdown HaCaT cell line (Figure 2E). This was further confirmed with a NBPF7 specific antibody (Figure 2E). In agreement with the Co-IP results, NBPF7 mRNA and protein levels were evaluated in the α-catenin-knockdown HaCaT cells (Figure 2B, 2C and Supplementary Figure 2). Moreover, immunofluorence analyses demonstrated co-localization of p65 and NBPF7 in the nucleus of α-catenin-knockdown HaCaT cells (Figure 2F). These results suggested that knockdown of α-catenin promotes the activation of NF-κB, and its subsequent interaction with NBPF7.

### Inhibition of NBPF7 decreases the proliferation of HaCaT cells

**Figure 3:**
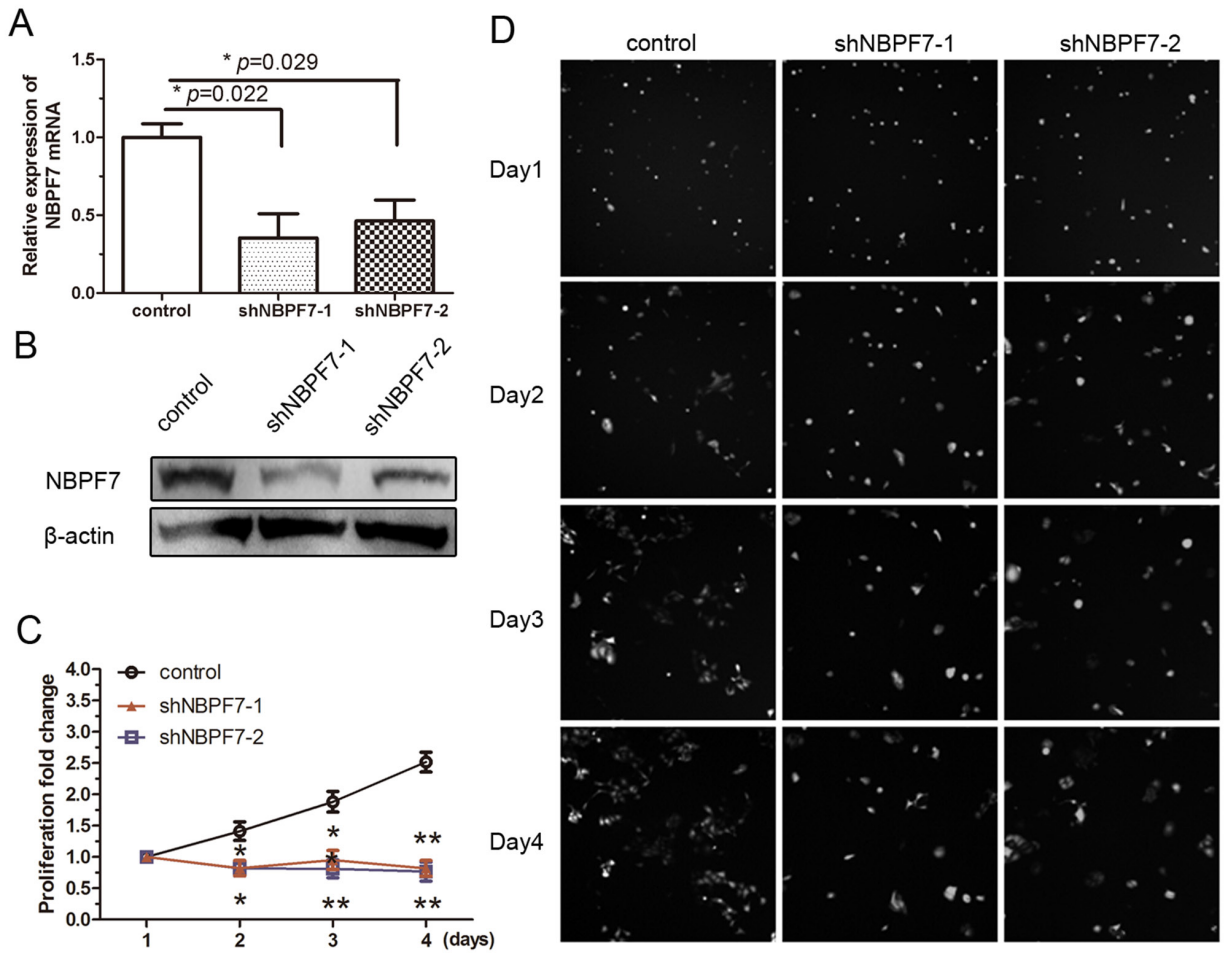
Stable knockdown of NBPF7 inhibited the growth of HaCaT cells **(A)** Expression levels of NBPF7 were examined by qRT-PCR in HaCaT cells. Experiments were performed three times. Data are presented as means ± SEM (*p*< 0.05, *p*< 0.01, independent t test). **(B)** Analysis of protein expression levels of NBPF7 in HaCaT cells infected with control (sh-control) or sh-NBPF-1 and -2 by using western blot. **(C)** and **(D)** Analysis of proliferation assay in HaCaT cells infected with control (sh-control) or sh-NBPF-1 and -2. Images magnification:100×. Values at the indicated time points were provided as the mean absorbance with an SEM (*p*< 0.05, *p*< 0.01, independent t test).

Given the upregulation of NBPF7, we tested the possibility that it may promote cell proliferation. To that end, we used shRNAs to stably silence NBPF7 in wild type HaCaT cells and examined the ensuing rate of cell growth. We found that the mRNA expression levels of NBPF7 were significantly decreased by NBPF7 shRNAs in HaCaT cells (Figure 3A). Similarly, western blot analysis showed decreased NBPF7 protein levels in these cells (Figure 3B). In contrast, the mRNA and protein levels of IκBα and p65 were not altered in HaCaT cells by NBPF7-knockdown (Supplementary Figure 3). Furthermore, knockdown of NBPF7 in HaCaT cells resulted in a decreased rate of cell growth, consistent with reduced proliferative capacity (Figure 3C and 3D). These results are consistent with the notion that NBPF7 is an important regulator of proliferation in keratinocytes.

### NBPF7 knockdown abolishes the enhanced proliferation of α-catenin knockdown HaCaT cells

**Figure 4:**
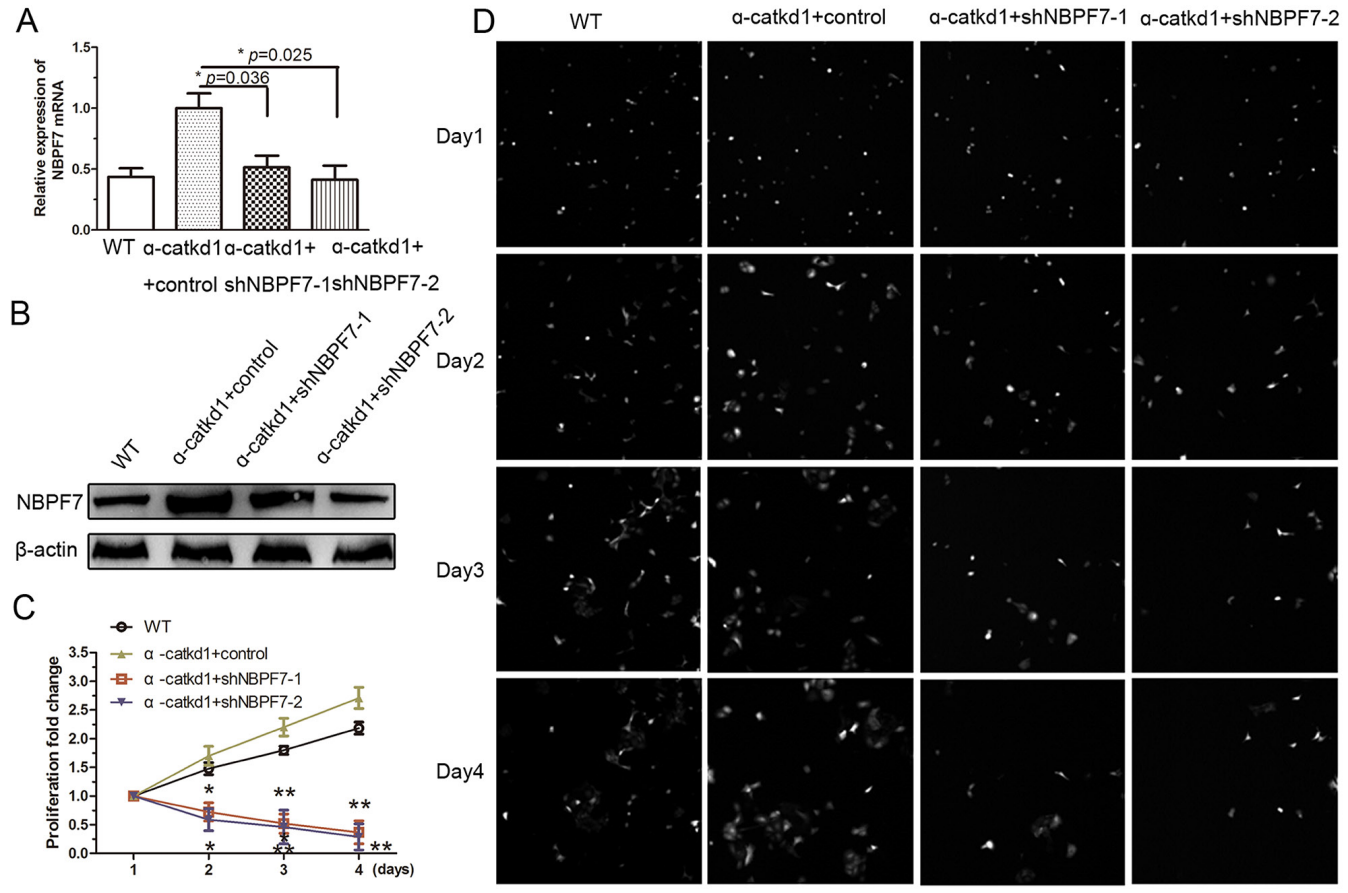
Silencing NBPF7 inhibited the enhanced proliferation of α-catenin knockdown HaCaT cells **(A)** mRNA levels of NBPF7 in α-catenin knockdown HaCaT cells after silencing NBPF7 with lentiviurs delivered shRNA as determined by qRT-PCR. Experiments were performed three times. Data are presented as means ± SEM (*p*< 0.05, *p*< 0.01, independent t test). **(B)** Protein levels ofNBPF7 in α-catenin knockdown HaCaT cells after silencing NBPF7 with lentiviurs delivered shRNA as determined by western blot. **(C)** and **(D)** Hyperproliferation of α-catenin knockdown HaCaT cell lines was rescued after stable knockdown of NBPF7. Images magnification:100×. Experiments were performed three times.

Our results thus far suggest that inhibition of NBPF7 might abolish the enhanced cell proliferation following loss of function of α-catenin in HaCaT cells. To test this possibility, we used the same shNBPF7 lentiviruses to infect α-catenin-knockdown HaCaT cells (α-catkd1). The mRNA (Figure 4A) and protein (Figure 4B) levels of NBPF7 were indeed upregulated in α-catenin-knockdown HaCaT cells, but were restored to the levels of wild type HaCaT cells by shNBPF7 lentiviruses. As anticipated, knockdown of NBPF7 rescued the cell proliferation rate of α-catenin-knockdown HaCaT cells to that of wild type HaCaT cells (Figure 4C and 4D). These results indicate that NBPF7 is a functional mediator of α-catenin signaling in the regulation of cell proliferation of keratinocytes.

## DISCUSSION

In this study, we presented the gene expression profile of HaCaT cells following deletion of α-catenin and found that NBPF genes were upregulated. We further showed that the NF-κB pathway was activated and NF-κB interacted with NBPF7 in α-catenin-knockdown HaCaT cells. Importantly, we found that inhibition of NBPF7 decreased the proliferation of HaCaT cells and abolished the enhanced proliferative capacity of α-catenin-knockdown HaCaT cells. These results indicated that NBPF7 plays key roles in the α-catenin signaling pathway that regulates cell proliferation of keratinocytes.

Although previous studies reported enhanced cell proliferation rates in α-catenin-deleted HaCaT cells [7, 19], the underlying mechanisms remained largely unknown. Because several actin-binding proteins have been found to modulate gene transcription [6], we set out to perform a whole-genome expression profiling analysis in α-catenin-knockdown HaCaT cells. We found that the gene expression levels of several members of the NBFP family were upregulated in the α-catenin-knockdown HaCaT cells, suggesting that the NBFP family members may play important roles in the suppression of cell proliferation by α-catenin.

Our further validation analyses showed that silencing α-catenin led to activation of the NF-κB pathway, accompanied with upregulation of NBPF7. The pattern of α-catenin-mediated destabilization of IκBα was also observed in breast cancer cell lines [11]. In contrast, loss-of-function of α-catenin has been shown after knockdown of IκBα protein only in TNF-α-treated cells. This TNF-α-treated model was different from HaCaT cells, because cancer cells within a tumor are usually exposed to TNF-α secreted by infiltrated macrophages or by the tumor cells themselves [11, 20]. It was reported that epidermal hyperproliferation triggered by the loss of α-catenin was due to loss of components of cell-cell adherens junctions [7]. Yap1 functions as an important effector via the Hippo signaling pathway in controlling organ size and proliferation of epidermal stem cells, whereas variations in the availability of catenin were shown to influence the functional status of NF-κB [21-23]. Our findings indicated that loss of α-catenin might be a mechanism by which the NF-κB pathway is activated in HaCaT cells.

Our results also suggest that NBPF plays key roles in the regulation of cell proliferation by the α-catenin/NF-κB pathways. In agreement with our findings, it was shown that NBPF genes were densely covered by many high-confidence ChIP-Seq peaks of NF-κB [11]. The NBPF genes exhibit a high variation of copy number, suggesting that this family of genes is likely to be involved in pathology of human diseases [24]. However, very little is known about the functions of the encoded NBPF proteins. In the human chromosome 1p36 region where NBPF is located, several tumor suppressor genes have also been identified, suggesting that NBPF might act as a tumor suppressor gene [14, 15, 25]. In support of this contention, previous study reported that NBPF1 inhibited the formation of soft agar colony of human colorectal cells [15]. In contrast, overexpression of NBPF12 has been found in several cancers including sarcomas [26], and non-small-cell lung cancer [27]. In this study, NBPF7 was found to be upregulated in a proliferation cell model. Moreover, we found that NBPF7 interacted with p65 and promoted proliferation of HaCaT cells. However, knockdown of NBPF7 did not alter the mRNA and protein levels of IκBα and p65 in HaCaT cells, suggesting that NBPF7 functions in parallel to or downstream of the NF-κB pathway in the regulation of cell proliferation by α-catenin circuit. Taken together, our findings suggest that this gene is involved in cell growth.

In summary, we found that NBPF genes were upregulated in α-catenin-knockdown HaCaT cells and NBPF7 through functional linkage with the NF-κB pathway promoted the cell proliferation of α-catenin-knockdown HaCaT cells. These results indicated that NBPF7 plays key roles in the α-catenin signaling pathway in the regulation of cell proliferation of keratinocytes.

## MATERIALS AND METHODS

### RNA oligonucleotides and cell culture

The shRNA for α-catenin and scramble control RNA oligonucleotides were chemically synthesized and purified with high-performance liquid chromatography by GenePharma (Shanghai, China). The sequences of the RNA oligonucleotides were as follows: shα-catenin-196 5’-CAGGUUACAACCCUUGUAATT-3’; shα-catenin-1254 5’-GGACCACGUUUCAGAUUCUTT-3’; shα-catenin-2555 5’-CAGCCAAGAACUUGAUGAATT-3’; Scramble: 5’-CAGUACUUUUGUGUAGUACAA-3’. RNA oligonucleotides were transiently transfected using Lipofectamine 2000 (Invitrogen, NY, US). HaCaT cell culture was performed as previously described [7].

### Stable knockdown of α-catenin with lentivirus-delivered shRNA

α-catenin knockdown in HaCaT cells was achieved by shRNA infection (Gipz ctnna1 lentiviral shRNA transduction starter kit, Open Biosystems/GE Dharmacon, CO, US) as previously described [7]. The shRNA targeting sequences were as follows: α-catenin-1, 5’- TTTGGTAGAGGCGACGTAG-3’; α-catenin-2, 5’-TTATTTGAGATGGAACAGG-3’. negative controls: GIPZ non-silencing lentiviral shRNA control. Lentivirus packaging and infection were performed according to standard protocols as recommended by the manufacturer.

### Gene expression profiling

Total RNA was extracted from HaCaT cells 3 days post-transduction of α-catenin RNA oligonucleotides using TRIzol reagent according to the manufacturer’s instructions (Invitrogen, NY, US). cDNA was synthesized using GoScript™ Reverse Transcription System according to the manufacturer’s instructions (Promega, A5001). Labeling of cDNA and hybridization to Affymetrix PrimeView human gene expression array was performed using GeneChip® Hybridization, Wash and Stain Kit (Cat900720, Affymetrix, Santa Clara, CA, US) in Hybridization Oven 645 (Cat00-0331-220V, Affymetrix) and Fluidics Station 450 (Cat00-0079, Affymetrix) according to the manufacturer’s instructions. Slides were scanned by GeneChip® Scanner 3000 (Cat00-00212, Affymetrix) and the data were analyzed with Command Console Software 3.1 (Affymetrix) using the default settings. Raw data were normalized by RMA algorithm with Gene Spring Software 11.0 (Agilent technologies, Santa Clara, CA, US).

### Western blotting

The following antibodies were used for western blot analysis: anti-NBPF15 rabbit polyclonal (1:500, 12848-1-AP, Proteintech, US), anti-NBPF7 goat monoclonal (1:500, sc-248056, Santa Cruz Biotechnology, US), anti-α-catenin mouse monoclonal (ab49105, Abcam, MA, US), anti-NF-κB (p65) mouse monoclonal (1:1000, Cat#6956, Cell Signaling Techology, MA, US), anti-IκBα mouse monoclonal (1:1000, Cat#4814, Cell Signaling Techology) and anti-β-actin mouse monoclonal (1:1000, Cat# A5441, Sigma-Aldrich, MO, US). Signals were developed with the enhanced chemiluminescence detection system (Pierce, Thermo Fischer Scientific, Bonn, Germany).

### Immunofluorescence

Cells were fixed in 4% paraformaldehyde for 15 min followed by blocking in PBS with 2.5% normal goat serum, 0.3% triton X100, and 2% bovine serum albumin for 30 min. Sections were incubated in primary antibodies against NBPF7 (1:100) and NF-κB (p65) (1:200) for 1 hour at room temperature. The nucleus was counterstained by DAPI and imaged using a Nikon eclipse E600 microscope with DP Manager Version 1.2.1.107 software.

### Quantitative reverse transcriptase-PCR analysis

Total RNA was extracted from cultured cells using TRIzol reagent (Invitrogen, NY, US) according to manufacturer’s instructions. 1 mg of RNA was employed to synthesize cDNA using the PrimeScript RT reagent kit perfect real time (TaKaRa, Dalian, China) or the miScript II RT Kit (Qiagen, Germany). Samples were run in triplicate and normalized to GAPDH. Primer sequences are as follows: α-catenin forward primer: 5’- GGGGATAAAATTGCGAAGGAGA-3’; reverse primer: 5’- GTTGCCTCGCTTCACAGAAGA-3’; NBPF7 forward primer: 5’- CTGTGGATTTGTGGGCTGAAG-3’; reverse primer: 5’- GGAGTGTTCGCTGGCTACAT-3’; GAPDH forward primer: 5’- ATTTGGTCGTATTGGGCG-3’; reverse primer: 5’- CTCGCTCCTGGAAGATGG-3’;

### Co-immunoprecipitation (Co-IP)

Co-immunoprecipitation studies were performed by lysing cells in RIPA buffer followed by pre-clearing with protein A/G agarose beads. Primary antibodies of interest (NF-κB p65 at 1:100) were added, and protein/antibody complexes were incubated overnight at 4°C, and captured on protein A/G beads. Samples were then eluted in laemmli buffer and analyzed by Western blot.

### Cell proliferation assay

Cells were plated at concentration of 2000 cell/well in 96-well plates in triplicate. The cells were monitored for cell proliferation with the Cellomics arrayscan VT1 (Thermo, MA, US) according to the manufacturer’s directions for 5 days. Cellomics scan was performed at each time point in 5 fields. Captured images were analyzed for cell counts.

### Statistical analysis

All data are expressed as the mean ± SEM of at least three separate experiments. The statistical significance between two experimental groups was indicated in the figures by asterisks, and comparisons were made using the Student’s t test. Calculated P-values less than or equal to 0.05 were considered to be of statistical significance. Data were analyzed with the PRISM software version 4 (GraphPad Software, CA, US).

## SUPPLEMENTARY MATERIALS FIGURES


