# Supplementary material for: NBPF7 promotes the proliferation of α-catenin-knockdown HaCaT cells via functional interaction with the NF-κB pathway
Source: Oncotarget. 2017 Jul 22;8(39):65800–8. doi: 10.18632/oncotarget.19480 (PMC5630373; doi:10.18632/oncotarget.19480)
Supplement: Supplementary file 1 [file oncotarget-08-65800-s001.pdf]

NBPF7 promotes the proliferation of α-catenin-knockdown HaCaT cells via functional interaction with the NF-κB pathway

SUPPLEMENTARY MATERIALS

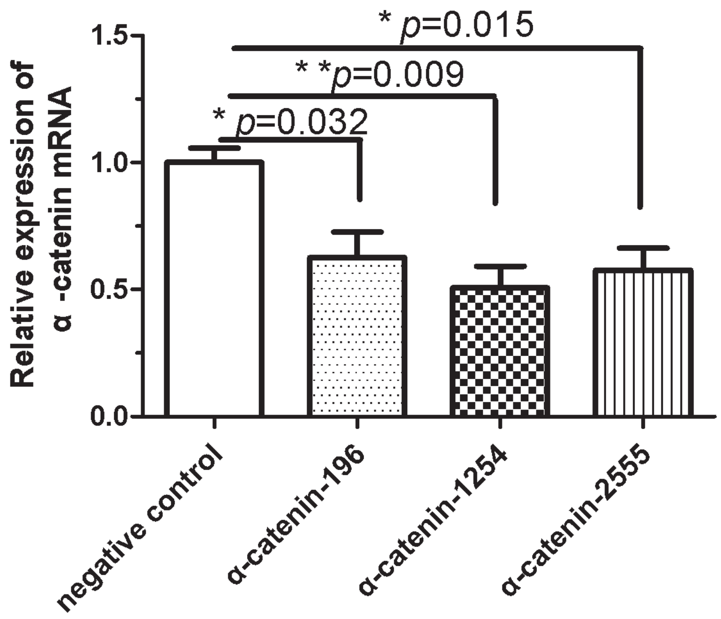

Supplementary Figure 1: qRT-PCR analysis showing α-catenin expression in HaCaT cells following transient transfection with RNA oligonucleotides (p< 0.01, independent t test).**

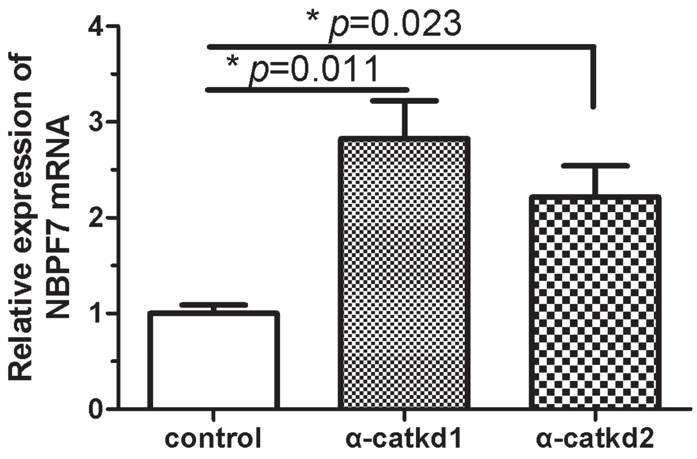

Supplementary Figure 2: qRT-PCR analysis showing NBPF7 expression in α-catenin-knockdown HaCaT cell lines (*p< 0.01, independent t test).

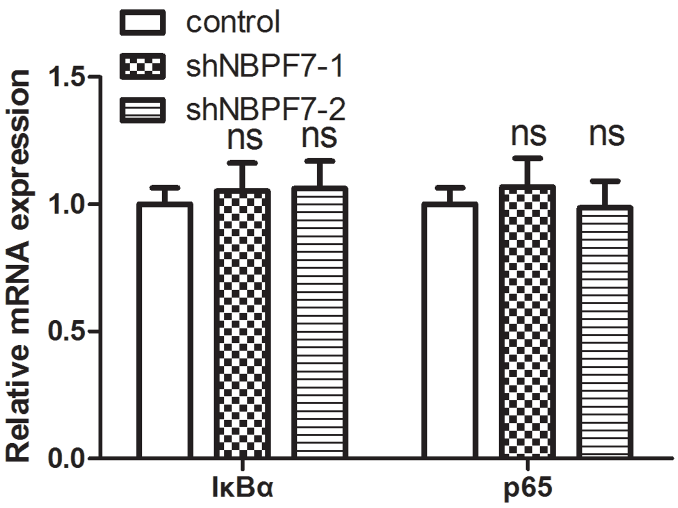

Supplementary Figure 3: qRT-PCR analysis showing IκBα and p65 expression in HaCaT cells stable knockdown of NBPF7 with lentivirus-delivered shRNA. Experiments were performed three times. Data are presented as means ± SEM ($p< 0.05$, independent t test).
